# Representation: raising awareness of opportunities and skill development with undergraduate dental students

**DOI:** 10.1038/s41415-022-4352-1

**Published:** 2022-06-24

**Authors:** Luisa Wakeling, Paul Blaylock, Matthew Harper, Miranda Trevor, Holly Murphy, Joe Barton, Heidi Bateman

**Affiliations:** 41415123984001 School of Dental Sciences, Faculty of Medical Sciences, Newcastle University, Newcastle upon Tyne, NE2 4HH, UK; 41415123984002 School of Medical Education, Faculty of Medical Sciences, Newcastle University, Newcastle upon Tyne, NE2 4HH, UK; 41415123984003 Newcastle University Students´ Union, Newcastle University, Newcastle upon Tyne, NE1 8QB, UK

## Abstract

Representatives for dentists are required for many governing committees, local and national, that contribute to many aspects of the profession related to politics, the workplace, education, or community-building. Developing skills as a representative can begin as an undergraduate in student representation systems as part of UK university governance structures. At one UK dental institution, there was a plan to explore the learning and skill development of current student representatives, review the training, identify any areas where there were gaps or where they should be strengthened, and consider whether a new training programme could be developed. Training gaps in the representation process and preference for peer mentoring in training were identified as students acknowledged learning 'on the job' through observation of experienced peers. Current representation training also fell short of highlighting the relevance to their future dental profession. Staff and students co-designed a bespoke programme of training to help students develop their representation skills, as well as align them to the development of professional skills which were determined to be relevant for their future dental career.

## Key points

- Outlines a selection of representation opportunities in the UK dental community.
- Explores professional skills developed by students through representation activities.
- Outlines a method for developing representation skills and the relevance of these.

## Introduction

This paper outlines representation opportunities in the UK dental profession and considers development of skills for dental students to support representation roles. Dental student opportunities in academic representation are described, together with a description of training that was co-designed and delivered by students and staff.

## Representation within the profession

Speaking or acting on behalf of another in representation is found throughout dentistry in various guises. The British Dental Association (BDA), as the trade union for dentists, primarily describes representation as involving elected individuals working to present the views of the electorate as the 'voice of members', thereby furthering their cause.^1^ This involves liaising with those who they represent and clearly articulating their views. The expectation is that positive outcomes will typically result for both the profession and patients.^2^

**Fig. 1:**
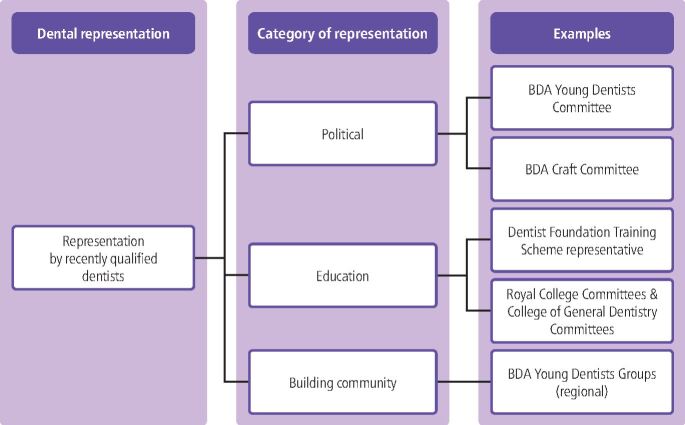
Representation opportunities for recently qualified dentists

**Fig. 2:**
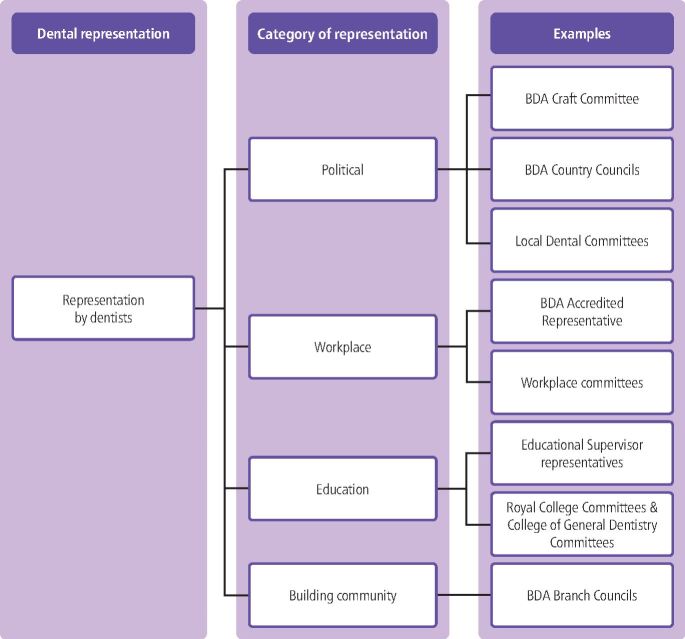
Representation opportunities for dentists during a career

The BDA is the best-known entity in UK dentistry which enables representation for dentists, with its constitutional structure separated into governance and representation.^1^ However, there are multiple other organisations and structures which support representation within dentistry. These are grouped into those most closely aligned with recently qualified dentists (Fig. 1) and established dentists throughout their career (Fig. 2), with examples provided. These examples have also been categorised based upon their primary focus: namely political, workplace, education, or community-building, although there is frequently cross-over between these aspects. This approach was developed as part of representation training delivered within this initiative, drawing upon elements of representation recognised by the BDA.^1^

Political representation takes place nationally by country within the UK, through county councils and by craft, to reflect the breadth of the dental workforce. Political representation aims to promote the cause of dentistry and dentists, including optimising NHS terms and conditions. Local representation includes local dental committees, which work to improve terms and conditions in their area. The BDA Students Committee includes two representatives from every UK dental school, offering an immediate opportunity for dental students to represent their peers nationally.^3^

Representation within an educational context includes: dental students on Student-Staff Committees (SSC) in dental schools; dental foundation training scheme representatives on Health Education England and deanery committees soon after graduation; and Royal College committees throughout training. These committees focus on optimising educational and training experiences for their electorate and promoting the cause of the areas they represent.

Workplaces offer a range of representational structures and committees. Roles such as the BDA Accredited Representative act in a trade union capacity to support employed dentists within their workplace.

The representation opportunity categorised as building community comprises mainly elected roles, where the priority is to support networking, support and social opportunities. There is significant overlap with other categories of representation. The British Dental Students Association serves this purpose, with its representatives organising social events for dental students^4^ but those same representatives sit on the BDA Students Committee delivering political representation. BDA Young Dentists Groups and branch councils include community-building representation regionally, in addition to organising educational events.

With many representation roles available, awareness of the need of representation, encouragement to participate and appropriate training is warranted. It has been recognised that diversity in representative roles must be improved, which includes greater involvement of newer members of the profession, who may require additional training.^5^

## Representation in dental education

**Fig. 3:**
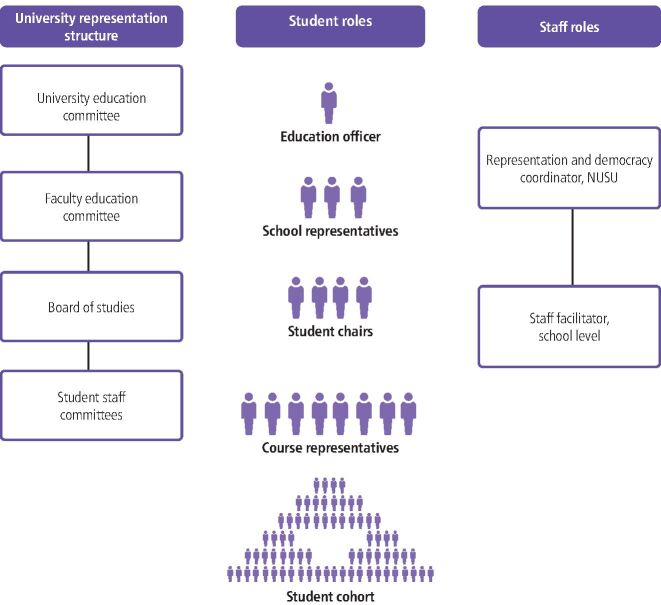
The structure of the Newcastle University's Academic Representation system including student roles and staff support

Higher education providers must actively engage students in the quality assurance of their learning experience.^6^^,^^7^^,^^8^The Newcastle University's academic representation system, overseen by Newcastle University's Students' Union (NUSU) is part of this quality assurance process that is supported through SSC meetings where student representatives in the School of Dental Sciences gather peer opinion on the quality of their programme, bring items for resolution and feedback actions to their cohort (Fig. 3). Student representatives also attend Board of Studies meetings to feedback on curricula, education initiatives and other regulatory processes. These in turn can be fed into overarching committees at faculty and university level. Student feedback is bidirectional within this structure. In any one academic year, student representatives can attend four student-led SSC meetings and eight staff-led Board of Studies meetings. Specific student representative roles within the Newcastle University system are outlined in Table 1.

**Table 1:** Description of roles in student representation

**Representation role**	**Outline of responsibilities**
Course representative	Gathers feedback from the student cohort about their learning experience on their degree programme
Student chair	Chairs SSC meetings where feedback is discussed with staff
School representative	Oversees student representatives and represents their degree programme at faculty and university level
Education officer	Represents all students at the university
Staff facilitator	At school level, supports student representatives in their roles
NUSU representation and research coordinator	Supports and provides training to students and staff facilitators across the university

When this development process began, new course representatives and student chairs received a tailored training lecture from NUSU and the dental school's staff facilitator where an overview of their role in representation was given with the following learning objectives:

- Understanding responsibilities as a course representative/student chair
- Being confident in engaging with your SSC
- Be able to effectively promote yourself as a course representative/student chair.

A Course Representative Handbook was also available from NUSU to provide an overview of the role.

An initiator to this project was a cursory analysis of topics brought for discussion to SSC meetings: a rich agenda of items more closely related to professional issues than typical problems encountered by students. Examples included matters arising around patient consent, patient safety and clinical governance within the dental hospital. The committee had therefore become a forum to improve the delivery of dental care within the environs of the dental school and as such, demanded a skill set to deal with such professional issues. Were students adequately prepared for this? Developing and demonstrating such skills are important as a student moves through their degree becoming a professional; extracurricular opportunities can provide additional spaces for sustained and deeper practise of these types of skills.^9^^,^^10^

## Methods

### Phase 1: student opinion on involvement with representation within the school

Dental students of the Newcastle University's School of Dental Sciences representation system were recruited to a semi-structured focus group which was audio-recorded and led by students employed through internships, experienced in facilitating focus groups. The transcript was coded by three independent members of the team (including the two student interns) and subsequently analysed by thematic analysis.^11^ A focus group was chosen to facilitate discussion and allow time for each student to describe their experience of involvement in representation. A topic guide was devised by the two student interns who used their experience and previous training in the representation system to focus the guide on exploration of professional learning opportunities facilitated by participation in representation. Individual open coding led to identification of themes that were agreed by all members of the team.

### Phase 2: review of training

The two student interns considered secondary data in the form of training material provided to student representatives: the NUSU training lecture slides and the Course Representative Handbook material was reviewed for content related to the development of professional learning opportunities.

### Phase 3: development and delivery of a new training programme

**Fig. 4:**
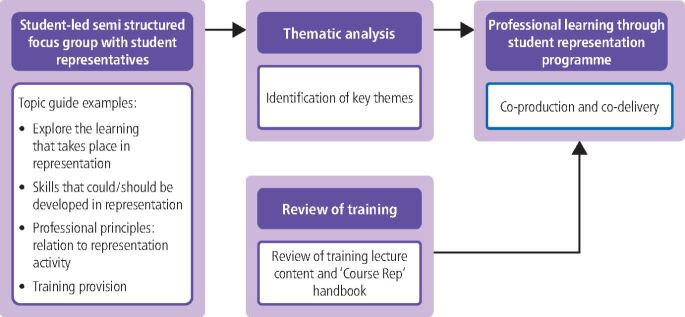
Exploration of learning opportunities in representation which led to co-design and development of a training programme

In response to findings in Phase 1 and Phase 2, a new programme of training was co-designed by the student interns, the dental school's staff facilitator and the NUSU representation and research coordinator. A series of meetings led to design and development of lesson plans for two workshops, creation of workshop activities and presentation slides, practice sessions and workshop implementation followed by debrief. Figure 4 outlines the approach to this work.

### Ethical consideration and funding

A University Research Ethics Committee ethical review application (no. 13512/2016) determined the project low-risk. Participants provided written consent to participate in the focus group and to be audio recorded. They could withdraw at any time. The project was supported through an internal university-wide Education Innovation Fund and a Faculty Educational Research and Development Project grant.

## Results

### Student opinion on learning opportunities and current training

Five students took part in the focus group (age range of 18-25 years). Participant role, sex and length of involvement in the Academic Representation system are shown in Table 2. Thematic analysis of the focus group transcript identified four overarching themes: responsibility; identity; training; and learning. Subthemes and descriptors (which outline content) and illustrative quotes are shown in Table 3. Themes are further described below, together with a selection of indicative quotes.

**Table 2:** Demographics of focus group participants

**Participant**	**Sex**	**Roles in representation system**	**Length of experience (years)**
1	M	Course rep, school rep	4
2	M	Course rep	1
3	F	Course rep, student chair	4
4	F	Course rep, student chair	4
5	F	Course rep	1

**Table 3:** Results from the focus group with student representatives following thematic analysis. Themes and subthemes together with a descriptor to outline the content and illustrative quotes are included

**Themes**	**Subtheme**	**Subtheme descriptor**	**Illustrative quotes of the subtheme**
Responsibility	Role in the representation system	Expectations of self and others	'We have to put work into it [rep role] and time' 'They expect us to bring it [student issue] up'
Identity	Identity of self	Personal progression and place in the system	'I've got a lot out of it' 'Being more integrated into the whole representation system…and committee process'
	Identity of others	Role of others including levels of engagement	'Some people will come to the occasional meeting…but aren't necessarily representing people' 'There is always going to be more people who do a lot more than others'
Training	Current provision and methods	Opinion on training and observation and discussion with peers	'Most training you get is on the job' 'We got to sit down with the 2nd years and see how they did it' 'Even the little debrief we had…after the SSC meeting. That was helpful'
	Gaps in training and learning	Challenges of the process, detail and context in training and suggestions for further training	'I had to go and look up what minutes and agenda means' 'I wish someone had gone "oh don't worry about it, it's not always like that"' 'Maybe if Phase 2 chairs were to train the first years'
Learning	Skill development	General skills such as time management, organisation, and confidence	'Became better at collecting information' 'Organise in advance' 'Try to find a solution'
	Representation system	Insight into the system and the importance of the student voice	'…not just the SSC but kind of going higher up to the board of studies and then kind of higher up again' 'Come across similar issues year on year' 'They might not actually really know what a student thinks' 'See things changing…they do actually listen'
	Observation of others and process	Having exposure to the process, observing peers and staff in committees	'…how they [peer] do chair work' 'I have noticed (anonymised staff) do that in Board of Studies' 'Going through the process helps you learn more'
	Professional learning	Consideration of best interests of those being represented, confidentiality and discretion, professionalism, alignment with General Dental Council principles	'You represent different groups of people and you kind of have to remain independent' '…where you actually have a very strong opinion on something, and you actually have to act very impartial because that's the best kind of most constructive way' '…remain professional' 'Confident bringing things up or raising concerns' '…you need to maintain the students' confidence in the SSC to make changes'

#### Theme: responsibility

Participants discussed their representation role responsibilities: ensuring preparation for meetings, conducting the role in the '*most professional way*' and supporting others in meetings. They also recognised the responsibility to others within the system and the importance of this in the success of the meeting:

- '*[As Chair,] have responsibility for your reps. I think it's just a little bit more of a leadership, communication kind of role*'.

#### Theme: identity

Examples related to the identity of themselves and others in the representation system, what they brought as individuals ('*how you represent your year*') and how their role meant they had a 'place in the system' which could be developed:

- '*I felt a bit out of place about bringing things up with senior members of staff but I feel like now it's quite a friendly atmosphere*'.

Participants talked about how they have changed with taking part and progressing through roles. Initial lack of perceived capability was recognised but also how this improved:

- '*[I] became more confident and couldn't imagine a couple of years ago chairing a meeting in front of very important, more educated people than me, but now I see I can do that'*.

#### Theme: training

There was no obvious negativity towards the current training; however, discussion revealed students attained most of their skills 'on the job' and by observing and working with others:

- '*I think ideally like I'd want to know a bit more when I first went into it but kind of going through the process makes you learn more*'.

Students faced challenges in mechanisms of feeding back to share meeting outcomes with their peers ('*it's just an issue to make it available for everyone*') and there was some confusion as to what should be shared:

- '*An individual came to me and asked me about a specific thing which I brought to the SSC on Wednesday and do I just tell that individual the result of it or do I spread it year wide?*'

Student chairs raised the challenge of writing an agenda and how poorly prepared they felt. Despite model agendas being provided, students wanted more support and recalled self-perceived inadequacy of the first agenda they produced. Participants suggested ways training could be improved by peer support and greater exposure to the system:

- '*Students sitting down with first years and going "this is how it works*" '
- '*I think the more times you do it and the more other meetings you go to as well, you just sort of as you go learn what works and what didn't work*'.

#### Theme: learning

Development of skills, such as time management, planning, communication, independence, problem solving, strategic judgement, collaboration and confidence were all described.

Learning about the representation system drew out insights including the importance of the student voice and it 'feeding upwards' (why staff would seek student opinion) and that this did lead to actual change:

- '*You see how the system works a little bit more and your place within the system and actually why they want student opinion. And why things that get brought up don't get changed and why certain things do get changed and what the purpose of the committee is as a whole really*'.

Learning about the role revealed the importance of observation of others and that they were also learning from observing staff in staff-led education meetings:

- '*When [anonymous] chairs the Board of Studies meeting, you sort of pick-up tips and advice as you go along*'.

Participants spoke of learning from earlier, less successful attempts at meetings. Students agreed that going through the process makes them learn but disliked the negative feelings when they began and refer to some early experiences as 'awful'. Conversely, students feel these difficult situations help them progress:

- '*I sort of learn on the job I guess, it's quite good to have that awful SSC meeting quite early, sort of like, get it out of the way. I can't imagine it would get any worse than that*'.

There were elements of learning that linked to professionally relevant contexts; working in the best interests of students and, for clinical year students, 'the patients'' best interests:

- '*Not everyone agrees with what you think, [we] should be honest. You also have to defend a statement if you think it's in the patient's best interest*'.

Participants also talked about understanding the confidentiality and discretion associated with representing someone's views, raising concerns and being impartial:

- '*It's quite interesting to be able to kind of adapt the way you speak about things and think about things depending on where you're placed in a particular situation*'.

When asked if they thought the professional principles set out by the General Dental Council (GDC)^12^ related to representation activity, the group were confident that most, if not all professional principles aligned to aspects of the representation process:

- '*I'd honestly say possibly all of them, you could apply it to everything. Like patients' interests first, we had the discussion about patient confidentiality, things like that*'
- '*Communication, complaints procedures, I mean that's basically what it is isn't it?*'

Some felt that their professional skills were developed directly when patient-centred discussions took place within the SSC forum. There was also a feeling that professional principles could be developed further by applying them to indirect situations not relating to patient care; in essence, those relating to professional dealings with student colleagues and staff.

### Gaps identified from the review of training material

This review found only brief mentions of the benefits of involvement in student representation, mainly focusing on achievements in influencing the educational experience rather than personal development. Training did not significantly encourage continued involvement in representation and as such, student turnout at meetings had a tendency to reduce as the year progressed. Nothing was mentioned about specific skill development and potential influence for future career.

### Co-creation of a training programme specific for dental students

From focus group findings and training review, potential opportunities to improve training were identified:

- Structured representation activities, such as agenda writing, that are interactive and include skills practice
- Involvement of current representatives in the training, for example, as mentors - a personal development and progression opportunity for them
- Increase recognition of professional skill development in representation
- Information about professional representation opportunities at different career stages.

A pilot programme was developed to supplement the training lecture. This was optional and limited to those with an active role in the representation system. A key design element was that training would be student-led and supported by staff.

Two interactive workshops were developed and spaced apart in the academic year to allow practical meeting experience between training workshops.

#### Workshop 1: student representation training

Designed to explore the meaning and importance of representation and the process required to enable effective representation. Each activity was underpinned by real examples from SSC meetings and the student lead for the workshop supported participants by sharing their own experiences, guiding discussion and activities.

#### Workshop 2: professional skills in representation

Students worked through actual SSC scenarios and aligned experiences with professional principles. The workshop highlighted opportunities for representation at different career stages. This was delivered by a dentist with experience in local and national committees. This was illustrated with examples from the speaker's own career and achievements secured through those representative structures.

### Pilot programme assessment and participant recognition

Participants were assessed after each workshop by a reflective piece of writing. Successful programme completion resulted in a Newcastle University Students' Union 'Professional learning through Representation Award'.

A short pilot-programme evaluation was disseminated to all participants. This highlighted appreciation of the interactive nature of the workshop and the opportunity to have open discussion of their experiences with students from other stages:

- '*Gave me an idea of where our representation roles can take us in the future. Also that our skills can be used outside of the university and dental school in a vast number of roles that we can make a difference*'
- '*I think I definitely benefited from the workshops and they advanced my understanding of the workings of the dental school and the larger student body as well*'.

## Discussion

Academic representation is an extracurricular activity that allows students to feedback on behalf of their peers to the institution about their learning experience and provides opportunities for the student voice to influence education.^8^ There is an acknowledged paucity in the dental literature on the benefits to students in engaging with these processes compared with other professional courses, such as medicine and nursing.^13^ Effective representation can be transformative by empowering voices and there is evidence that effective student representation not only benefits the students involved, but also education providers and society.^14^ Where dental students are discussing issues around patient care alongside their curriculum, this also supports the requirements outlined as part of the GDC's *Standards for education*^15^ where education providers should attain multi-source feedback (including student feedback) on quality assurance aspects of the programme. As well as this, education providers should have a variety of mechanisms to ensure concerns about patient safety aspects can be raised.

The skills involved in representation and the act of representation in itself are highly valuable post-graduation, during a dental career.^1^ Through developing an understanding of the art of representation, a dentist can help to support and represent colleagues in their dental practice or other workplace. They can also ensure that systems are in place to enable the views of patients to be adequately represented, such as through a patient forum, which can enhance service development and outcomes.^16^ Other skills which are developed through being a representative, such as communication skills and leadership skills, bring additional benefits and opportunities to progress throughout careers.^12^

At a national, regional or local level, using these skills to become involved in political representation, or representation in an educational setting, can yield powerful results for the profession and oral health.^1^ Through influencing policy, these undertakings will benefit many dentists, frequently including the representative themselves. Representation opportunities exist via trade unions such as the BDA and also Royal Colleges, learned societies and other representative structures and networks.

These representative structures are not always aligned with the demographics of the population being represented and there are frequently gaps.^17^ Through supporting the next generation of dental students to gain representation skills, it is hoped that the numbers and diversity of dentists involved in such representational structures will increase in future, thereby improving that representation. It is also important to acknowledge that those students who take part in representation and moreover, the training programme, are likely to be actively engaged students. Widening access to the training and encouraging more student voices to take up representation roles will be an important commitment going forward to ensure diverse voices are heard and opportunities afforded to more.^18^

Within Newcastle University School of Dental Sciences, the workshops have now been delivered for three consecutive academic years with a total of 15 students completing the award. With the COVID-19 pandemic, workshops moved to a similar but online format in the academic year 2020-2021, which complemented the fact that all SSC meetings were held online. Students who have completed the award also now take a role in leading the training programme.

Workshop participants have progressed in their roles in the representation system from course representative to student chair and school representative. They have also moved into other roles including equality, diversity and inclusion representatives. Participants have also gone on to achieve NUSU's 'Academic Rep' Awards and 'Pride of Newcastle' Awards. Other outcomes have included dental graduates taking on representation roles during their foundation year, including involvement in the Deanery Representation Programme and the local BDA committee.

### Limitations

A limitation of this work was that only one focus group, with five participants, took place. Participants had different levels of experience, ranging from only a year in a representation role to four years of experience. While this divided the group demographic, it did mean that there were perspectives from those who had recent training and those who had more experience to reflect on the applicability of the training.

### Future directions and application

The programme is available to those who wish to further develop their skills. A future direction would be to include all student representatives with an overall aim to enhance engagement and quality in representation. Motivation for undergraduates to become involved in representation can include many reasons, such as to get to know and help people, personal gains and opportunities to make their CV stand out.^7^^,^^8^^,^^18^ Further exploration of this would be useful, along with evaluating the direct relevance of this training programme to future practice.

## Conclusion

By exploring the learning students experienced by taking part in representation and identifying training needs, we designed a bespoke programme to help develop representation skills and understanding of future opportunities.
